# The First Randomised Trial of Male Circumcision for Preventing HIV: What Were the Ethical Issues?

**DOI:** 10.1371/journal.pmed.0020287

**Published:** 2005-10-25

**Authors:** Peter Cleaton-Jones

## Abstract

Cleaton-Jones, chair of the ethics committee that approved the trial of circumcision for preventing HIV, shares with us the discussions that the committee had ahead of granting approval.

In the November issue of *PLoS Medicine*, Auvert and colleagues report the first randomised controlled trial of circumcision for preventing HIV infection [1]. In the study, 3,274 uncircumcised men, aged 18–24 years, were randomised to a control or intervention group, with follow-up at three, 12, and 21 months. Circumcision was offered to the intervention group immediately after randomisation and to the control group at the end of the follow-up (only men who wished to be circumcised were eligible for the trial). There were 20 HIV infections in the intervention group and 49 in the control group, corresponding to a relative risk of 0.40 (95% confidence interval, 0.24–0.68; *p* < 0.001). The relative risk was unchanged when controlled for behavioural factors, including sexual behaviour, condom use, and health-seeking behaviour, suggesting that male circumcision provides a degree of protection against acquiring HIV infection.

Prior to conducting the study, the study proposal (protocol study number M020104) was reviewed in January 2002 by the Human Research Ethics Committee (Medical), which is the institutional review board (IRB) of the University of the Witwatersrand in Johannesburg, South Africa, and which I chair. The committee decided to approve the study, and in this article I discuss how we came to our decision.

## Background to the Committee

The committee was formed in October 1966 soon after the seminal paper by Beecher on ethics and clinical research was published [2]. It has functioned continuously, ever since, according to local [3] and international [4] research ethics guidelines. The committee has United States federal-wide accreditation (FWA 0000715), and is one of the few IRBs outside the US that has had a site quality assurance visit by a team from the US Office of Human Research Protections (the visit was in 2002, headed by Deputy Director Melody Lin).

At the time when we reviewed Auvert and colleagues' protocol, there were 27 members, 12 women and 15 men, from diverse ethnic backgrounds. Twenty-four members were from the University of the Witwatersrand, of whom 16 had medical or scientific expertise and eight had nonmedical backgrounds in law, social work, ethics, and psychology. There were three independent members (with backgrounds in education, nursing, and religion). Seven members of the committee had, or were obtaining, postgraduate qualifications in applied ethics up to doctoral level. Over half the members had at least ten years of experience—the maximum was 28 years—and three members served, or had served, on the IRBs of the Medical Research Council and Human Sciences Research Council in South Africa. All but three were born in South Africa.

## Importance of the Study

There were four reasons why the committee believed that the proposed study had local importance, and that it should be conducted urgently. First, the proposal dealt with a serious infection with a seroprevalence in 2002 of 26.5% among pregnant women, according to a national government survey [5].

Second, in 2002, government policy concerning HIV was to provide condoms, safe-sex counselling, and voluntary HIV testing after counselling—but no antiretroviral treatment to those infected. Such treatment was only available from government health centres, starting in 2004 [6]. In other words, at the time when we considered the study protocol, the public-sector standard of care did not include antiretroviral medication.

Third, anecdotal evidence at the time when we considered this proposal suggested that circumcision might be protective against heterosexual acquisition of HIV in men, but firm evidence was lacking. A year later, a meta-analysis of published studies concluded that while epidemiological evidence was supportive, the outcome of randomised controlled trials would be important in determining the value of circumcision [7].

Finally, in many African cultures in South Africa, initiation into manhood is accompanied by circumcision performed by a “traditional surgeon”. In recent times, because of high mortality from haemorrhage, infections, and dehydration [8], many African men now opt for circumcision in adult life by a medical practitioner.

## Ethical Considerations

We were, therefore, convinced that the study was important, and we then went on to consider whether it would be ethical. There were four guiding principles in our ethical deliberations.

### Autonomy

Autonomy was respected through a written informed-consent process, with verbal translation into vernacular if needed. This process also fulfilled a local legal requirement, since informed consent to participate in research is entrenched in the Bill of Rights of the South African Constitution [9]. No false promises were made that might have influenced a person's decision to participate.

### Beneficence

All participants would receive their desired circumcision. They would also have repeated medical examinations and counselling at each follow-up visit. Such counselling involved education about safe-sex practices and the benefits of being tested for HIV. Participants who wished to know their HIV status were referred for an HIV test.

One criticism that has been levelled at studies of HIV in South Africa is that not all participants are routinely informed of their HIV status. Our committee understands the stigmatisation faced by people in South Africa who are HIV positive [10], and so we accepted the right of individuals in the study to choose whether or not to be tested, once they had been counselled.

If during the study a participant became infected with syphilis, or any other sexually transmitted disease for which treatment was provided at government health centres, the participant would be referred to the nearby health centre to receive treatment.

Beneficence was also allowed for through scheduled interim analyses, with results provided to a three-person data safety and monitoring board in order to stop the study as soon as statistically significant results had been found (see below).

### Non-maleficence

The study's proposed participants were to be only those who wished to be circumcised. All participants would receive their desired procedure, half immediately and half at the end of the study, so there would be no withholding of the operation. A potential source of disadvantage would be if there was a selection process before enrolment, for example, testing for HIV and accepting only people who were HIV negative into the study. The researchers had anticipated this, and all who wished to enrol were accepted—adjustment for HIV status at enrolment was through a large sample size and statistical analysis.

A second concern of the researchers was that the results of the tests for HIV and herpes simplex virus should not be known by the researchers in order to keep them “blinded” and, hence, minimise bias. Because voluntary testing for these viruses was offered at each follow-up, the researchers' concern was accepted by the committee.

No treatment would be provided for HIV or herpes simplex virus infection, according to government policy. The Declaration of Helsinki recommends provision of treatment for a disease being studied, and so our committee debated at length the issue of whether the researchers had an ethical duty to provide treatment. We concluded, as put forward by Benatar [11], that a moral judgment had to be made considering local conditions, the short period of the study (which was less than the 5–7 years considered appropriate at the time to reach a clinical need for antiretroviral treatment), and the finances involved. In 2002, the South African government was not offering treatment for these two infections, and so we agreed that it would be ethical for no treatment to be provided to the study's participants.

### Justice

Justice was adhered to by ensuring that the potential participants were recruited from disadvantaged groups who might not have had ready access to medical circumcision.

## Interim Analyses

The first interim analysis showed no statistically significant effect. The second, at 12 months, did show such an effect, and so the data safety and monitoring board recommended that the study be stopped—and the committee agreed. At this stage, the researchers amended the study to allow HIV, herpes simplex virus, and syphilis results recorded in the study to be given to those participants, after counselling and informed consent, who had not previously wished to know.

By then the government policy had changed to providing antiretroviral therapy. The researchers undertook to do their best to provide HIV treatment for up to five years to those participants for whom such treatment was clinically indicated, and the committee approved this course of action.

## Conclusion

This randomised trial presented a challenge to the IRB to combine general principles of research ethics with local conditions to permit a very important study to be done. Functioning, as the IRB does, in a developing country environment requires knowledge of local cultures, resources, and services. This is not always understood nor accepted by IRBs and researchers who operate in resource-rich environments. The fact that circumcision is so clearly protective should benefit many men, particularly those in countries with a high HIV prevalence.

## Abbreviation

IRB: institutional review board
